# Supplementary material for: Transgenerational deep sequencing revealed hypermethylation of hippocampal mGluR1 gene with altered mRNA expression of mGluR5 and mGluR3 associated with behavioral changes in Sprague Dawley rats with history of prolonged febrile seizure
Source: PLoS One. 2019 Nov 11;14(11):e0225034. doi: 10.1371/journal.pone.0225034 (PMC6844483; doi:10.1371/journal.pone.0225034)
Supplement: S1 Table — (PDF) [file pone.0225034.s001.pdf]

# Transgenerational deep sequencing revealed hypermethylation of hippocampal mGluR1 gene with altered mRNA expression of mGluR5 and mGluR3 associated with behavioral changes in Sprague Dawley rats with history of prolonged febrile seizure

**Oluwole Ojo Alese[1*], Musa V. Mabandla [1]**

[1]Department of Human Physiology, College of Health Sciences, University of Kwazulu-Natal, South Africa

[*]Corresponding author:

E-mail address: alese44@yahoo.com

Department of Human Physiology, College of Health Sciences, University of Kwazulu-Natal, 4000, South Africa.

inqaba biotec

Inqaba Biotechnical Industries (Pty) Ltd
P.O. Box 14356, Hatfield 0028, South Africa
Tel: 012 343 5829
Fax: 012 343 0287
E-mail: info@inqaba.com

# SYNTHESIS REPORT

31 Oct 2016

Client Detail:

Oluwole Alese
University of KwaZulu-Natal, School of Laboratory Medicine Medical Sciences
Human Physiology, 2 F-409, University Road
Westville Campus
Durban
320
South Africa

| | | | | | |
|---|---|---|---|---|---|
| **Name:** | Grm5 gene F | | **Barcode:** S287E | **Length:** 20 bases | |
| **Sequence:** | TCCAGCAGCCTAGTCAACCT | | | | |
| **OD** | 12.0061 | **MW min \ max** | 6021.4\6021.4 | **5' Mod** | None |
| **nmoles** | 58.37 | **GC % min \ max** | 55\55 | **3' Mod** | None |
| **Tm min \ max** | 62.45\62.45 | | | **Purification** | Standard |
| **For a 100 µM stock solution add** 583.67 **µl water or buffer** | | | | | **PAGE QC Image >>** |
| **Comments** | | | | | |

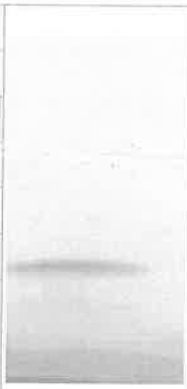

| | | | | | |
|---|---|---|---|---|---|
| **Name:** | Grm5 gene R | | **Barcode:** S287F | **Length:** 20 bases | |
| **Sequence:** | CAGATTTTCCGTTGGAGCTT | | | | |
| **OD** | 14.1588 | **MW min \ max** | 6113.8\6113.8 | **5' Mod** | None |
| **nmoles** | 69.3 | **GC % min \ max** | 45\45 | **3' Mod** | None |
| **Tm min \ max** | 58.35\58.35 | | | **Purification** | Standard |
| **For a 100 µM stock solution add** 693.04 **µl water or buffer** | | | | | **PAGE QC Image >>** |
| **Comments** | | | | | |

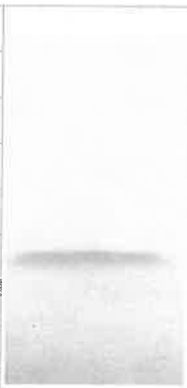

| Name: | Grm3 gene F | | | Barcode: S2880 | Length: 22 bases |
|---|---|---|---|---|---|
| Sequence: | CGCTCTCCTAATCTCCCTCTGG | | | | |
| OD | 14.3146 | MW min \ max | 6572.9\6572.9 | 5' Mod | None |
| nmoles | 71.39 | GC % min \ max | 59.09\59.09 | 3' Mod | None |
| Tm min \ max | 66.4\66.4 | | | Purification | Standard |
| For a 100 µM stock solution add 713.95 µl water or buffer | | | | | PAGE QC Image >> |
| Comments | | | | | |

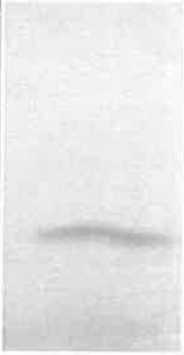

| Name: | Grm3 gene R | | | Barcode: S2881 | Length: 19 bases |
|---|---|---|---|---|---|
| Sequence: | CTCCTCTTCTCTTATCAGG | | | | |
| OD | 14.5863 | MW min \ max | 5680.7\5680.7 | 5' Mod | None |
| nmoles | 83.02 | GC % min \ max | 47.37\47.37 | 3' Mod | None |
| Tm min \ max | 58.01\58.01 | | | Purification | Standard |
| For a 100 µM stock solution add 830.18 µl water or buffer | | | | | PAGE QC Image >> |
| Comments | | | | | |

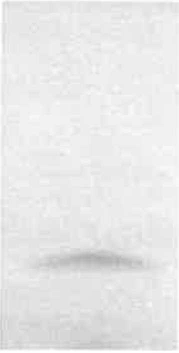

## RECOMMENDATIONS FOR HANDLING AND STORAGE OF OLIGOS

- Lyophilized oligo pellets might become displaced from the bottom of the tube during shipment. Briefly centrifuge each tube before opening to prevent the loss of the pellet.
- Prepare stock solution of oligos (e.g. 100 uM = 100 pmole per ul) preferably with a sterile buffered solution such as TE (10 mM Tris, pH 7.5 to 8.0, 1 mM EDTA). If sterile distilled water is used, make sure that the pH is above 7.0 since acidic solutions favours oligo depurination and subsequent loss of activity.
- Working solutions might be diluted from the stock solution with sterile, nuclease-free water to prevent inhibition of enzymatic reactions (e.g. PCR) by EDTA.
- Store the oligos as concentrated stock solution or lyophilized at -20° C.
- Avoid frequent freeze-thaw cycles by dividing the stock solution into smaller aliquots for long term storage and to prevent accidental contamination.
- Dye-modified oligos are light sensitive and should always be stored in the dark.
- Re-suspend modified oligos preferably in a slightly basic solution (i.e., TE at pH 8.0). However, Cy dye modified oligos are best kept at pH 7.0 at -20° C.
- Preferably store the modified oligos as dried aliquots at -20° C.
